# Detection of amastigotes and histopathological alterations in the thymus of *Leishmania infantum*‐infected dogs

**DOI:** 10.1002/iid3.285

**Published:** 2020-03-24

**Authors:** Aurea V. A. da Silva, Tainã L. de Souza, Fabiano B. Figueiredo, Artur A. V. Mendes, Luiz C. Ferreira, Camila P. B. Filgueira, Patricia Cuervo, Renato Porrozzi, Rodrigo C. Menezes, Fernanda N. Morgado

**Affiliations:** ^1^ Laboratório de Pesquisa em Leishmanioses IOC/FIOCRUZ Rio de Janeiro Brasil; ^2^ Laboratório de Biologia Celular, Instituto Carlos Chagas Fundação Oswaldo Cruz Curitiba Paraná Brasil; ^3^ Laboratório de Pesquisa Clínica em Dermatozoonoses em Animais Domésticos INI/FIOCRUZ Rio de Janeiro Brasil; ^4^ Serviço de Anatomia Patológica INI/FIOCRUZ Rio de Janeiro Brasil

**Keywords:** canine visceral leishmaniasis, clinical signs, extracellular matrix, histopathology, *Leishmania infantum*, parasite load, thymus

## Abstract

**Introduction:**

In canine visceral leishmaniasis (CVL), lymphopenia, and the disorganization of lymphoid organs such as spleen and lymph nodes have been demonstrated. However, the involvement of thymus in CVL has not been evaluated so far. Herein, we investigated whether the thymus can be colonized by *Leishmania infantum* in naturally infected dogs.

**Methods:**

Thymus were obtained from 16 of 58 dogs and samples of this organ were submitted to immunohistochemistry for laminin and fibronectin detection, histopathology, in situ hybridization and polymerase chain reaction (PCR) targeting the gene ITS‐1 for *Leishmania* and sequenced. Samples of spleen, skin and popliteal lymph nodes were collected and submitted to immunohistochemistry and parasitological culture followed by multilocus enzyme electrophoresis.

**Results:**

*L. infantum* was identified in all dogs. DNA and amastigote forms of *Leishmania* were detected in the thymus from 16 dogs by PCR and in eight by immunohistochemistry. Besides thymus, parasites were detected in spleen, lymph nodes, and skin. A granulomatous or pyogranulomatous thymitis was observed in eight dogs associated to intact amastigotes forms of this parasite. Fibronectin deposition in thymus was higher in dogs with more clinical signs.

**Conclusions:**

These results demonstrate that the thymus of dogs can be parasitized by *L. infantum*, which may generate inflammatory reactions leading to alterations in thymic microarchitecture.

## Graphical abstract

The thymus of dogs can be parasitized by *L. infantum*. Infection generates inflammatory reactions leading to thymic alterations. Thymic extracellular matrix alterations are associated with disease worsening in canine visceral leishmaniasis

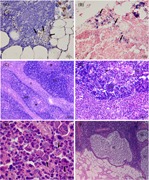

## 1 INTRODUCTION

Visceral leishmaniasis (VL) is a neglected zoonotic disease caused by the protozoan *Leishmania infantum* in the Americas.^1^ Dogs play an important role as domestic reservoirs of VL in the urban space since they harbor parasites available for sand fly vectors even in the healthy skin.^2^ *L. infantum* infects macrophages and can spread systemically to all canine tissues leading to the appearance of multiple clinical signs.^3^ Among the most commonly infected organs are the spleen and lymph nodes, which are essential to antigen presentation and the adequate development of a specific immune response.^4^ The disruption of splenic microarchitecture has been described in canine VL,^5^ as well as a pyogranulomatous or granulomatous inflammatory infiltrate, high parasite load and hypertrophy.^6^, ^7^ These alterations in the spleen are associated with the deposition of collagen, the impairment in cytokine messenger RNA expression and uncontrolled parasite growth in infected dogs.^5^, ^8^, ^9^, ^10^

In the lymph node, another secondary lymphoid organ, higher parasite load and higher expression of inducible nitric oxide synthases have been reported in symptomatic dogs when compared with asymptomatic animals.^11^ Histopathological alterations such as pyogranulomatous or granulomatous inflammation, hypertrophy, and hyperplasia of cortical and medullary zones^7^, ^12^, ^13^ and collagen deposition have been reported.^8^ Both, spleen and lymph nodes are important sites for the development of the specific immune response^13^ once they receive the majority of mature T lymphocytes from the thymus and form a delicate compartmentalization based on chemotaxis and an intrinsic antigen presentation.^9^, ^14^, ^15^

Although the association between parasitism and histological lesions has been frequently described in the spleen and lymph node of dogs infected by *L. infantum*, little is known about the parasite occurrence and pathogenesis in the thymus in these cases. The thymus is a primary lymphoid organ that plays a role in T‐cell homeostasis maintenance, and its product can dictate the immunological profile of individuals.^16^ In the thymus, thymocytes initiate the genic recombination and expression of their TCR receptors and CD4^+^ and CD8^+^ surface molecules. Another extremely important function of the thymus is the positive and negative selection of T cells. In this process, macrophages at the corticomedullary junction remove, by phagocytosis, T lymphocytes that recognize both self‐major histocompatibility complex molecules and self‐antigens.^17^, ^18^ The extracellular matrix compounds such as laminin and fibronectin are essential to promote thymocyte maturation based on cellular migration from cortical to the medullar region and then to the periphery. After maturation, naïve T cells leave the thymus, reaching the bloodstream and populating the secondary lymphoid organs. However, lymphopenia and failure in cellular immune response have been described in infected dogs.^19^ In this context, the present study aimed to evaluate the occurrence of *L. infantum* in the thymus of naturally infected dogs, and to describe the inflammatory infiltrate, the microarchitecture alterations and the deposition of extracellular matrix components (laminin and fibronectin) in this organ associated with parasitism. Herein, we observed infection by *L. infantum* in the thymus of all studied dogs, as well as an association between thymic microarchitecture alterations and disease‐worsening.

## 2 MATERIALS AND METHODS

### 2.1 Ethical statement

The animals included in this study were *L. infantum*‐naturally infected dogs that were destined for euthanasia as recommended by the Brazilian Ministry of Health polices. All dog owners had provided formal written consent. The samples were collected during necropsies conducted by veterinarians from the Laboratório de Pesquisa Clínica em Dermatozoonoses em Animais Domésticos (LAPCLIN‐DERMZO–INI/FIOCRUZ). This study was approved by the Ethics Committee on Animal Use of the Oswaldo Cruz Foundation (CEUA/FIOCRUZ; Permit Number: LW‐54/13) and performed according to Brazilian Law 11794/08 and the Brazilian Society of Laboratory Animal Science.

### 2.2 Animals

A descriptive study was conducted using a nonprobabilistic sample of dogs from Barra Mansa (22°32′25.19″S and 44°10′35.33″W), Rio de Janeiro State, Brazil, a VL endemic area.^20^ These dogs tested seropositive for anti‐*L. infantum* antibodies by a rapid immunochromatographic test (Dual Path Platform; Bio‐Manguinhos, Rio de Janeiro, Brazil) and by enzyme‐linked immunosorbent assay (Bio‐Manguinhos). The tests were performed by public health services responsible for the VL surveillance and control program of the state of Rio de Janeiro, with the permission of the owners.

The seropositive dogs were sent by the local authorities (Municipal Health Department of Barra Mansa) to be euthanized at Instituto Nacional de Infectologia Evandro Chagas (INI), Fundação Oswaldo Cruz, Brazil. Euthanasia procedure was performed according to recommendations of the Brazilian Ministry of Health for the control of VL. From the 58 animals necropsied, we were able to recover enough thymus samples from only 16 dogs, which were included in the study.

### 2.3 Clinical evaluation

The animals were submitted to a physical examination that consisted of the inspection of the skin, ocular, and nasal mucosae, as well as palpation of the superficial lymph nodes and organs. The following clinical signs were considered compatible with VL: low body score, cachexia, apathy, pale mucosae, regional or generalized lymphadenomegaly, splenomegaly, hepatomegaly, skin ulcers, furfuraceous desquamation of skin, onychogryphosis, local or generalized alopecia, and keratoconjunctivitis.^3^, ^21^ According to the clinical signs compatible with VL, the dogs were classified into three groups: without clinical signs, with few clinical signs (one to three clinical signs) and with many clinical signs (more than three clinical signs).^22^

### 2.4 Sample collection

After physical examination, the dogs were euthanized using 1.0 mL/kg of intravenous Thiopental 1.0% (Thiopentax Cristália). After the detection of an absence of corneal reflex induced by deep anesthesia, 10 mL of intravenous potassium chloride 19.1% (Isofarma) were administered. At necropsy, thymus samples were fixed in 10% neutral formalin and processed routinely for embedding in paraffin for immunohistochemistry, in situ hybridization (ISH), histopathology, and Polymerase chain reaction PCR. Additionally, samples of spleen, skin and popliteal lymph nodes were collected aseptically, immersed in sterile saline, and submitted to parasitological culture followed by multilocus enzyme electrophoresis (MLEE) for *Leishmania* species identification.

### 2.5 Parasitological culture for isolation and identification of *Leishmania* species

The samples were seeded onto biphasic NNN/Schneider's Insect Medium (Sigma‐Aldrich Co, St Louis, MO) containing 10% fetal bovine serum and incubated at 26 ± 28°C^23^ for at least 30 days. The *Leishmania* promastigotes, isolated from tissues, were further identified at the species level by MLEE using five enzymatic systems according to protocols previously described.^24^

### 2.6 Histopathological evaluation

The sections (5‐µm‐thick) were stained with hematoxylin and eosin and examined by light microscopy (Nikon Eclipse E400; Tokyo, Japan). The cortical area (mm^2^), the medullary area (mm^2^) of tissue and the cortex:medulla ratio were analyzed using the ImageJ software (NIH).

### 2.7 Detection and quantification of amastigote forms of *Leishmania* spp. by immunohistochemistry

The slides were submitted to deparaffinization, rehydration, blocking of endogenous peroxidase, antigen retrieval, blockade of nonspecific protein binding, and incubation with polyclonal rabbit anti‐*Leishmania* serum diluted 1:500 following a previously described protocol.^25^ A polymer‐based detection system (HiDef Detection HRP Polymer System; Cell Marque, Rocklin, CA) was used for the detection of amastigote forms of *Leishmania* spp. according to the manufacturer's recommendations.

For the evaluation of the parasite load in the thymus, macrophages parasitized with amastigote forms of *Leishmania* spp. were quantified using a 1‐mm^2^ optical grid and a manual cell counter. The cells were counted in five fields at ×400 magnification in the most parasitized areas of the sections. The mean number of parasitized macrophages was calculated and parasitism was classified as absent, mild to moderate (0.2‐10 parasitized macrophages), and intense (more than 10 parasitized macrophages).

### 2.8 Detection of laminin and fibronectin by immunohistochemistry

After deparaffinization, rehydration, blocking of endogenous peroxidase, antigen retrieval, blockade of nonspecific protein binding (0.4% bovine serum albumin; Sigma‐Aldrich Co), the specimens were incubated with the primary antibodies directed to laminin and fibronectin (Abcam), followed by incubation with anti‐rabbit secondary antibody (Abcam) and streptavidin‐horseradish peroxidase. Aminoethylcarbazole (AEC kit; Invitrogen) was used as the substrate–chromogen system and the slides were counterstained with Mayer's hematoxylin (Sigma‐Aldrich Co). The slides were examined under a light microscope (Nikon). Laminin and fibronectin deposits were quantified using the ImageJ software (NIH) and we analyzed at least 10 pictures/microscopy field. The results were expressed as area fraction (representative of the percentage of the stained area).

### 2.9 *Leishmania* characterization by PCR

The *Leishmania* present in the thymus were obtained by PCR the internal transcribed spacer‐1 (ITS‐1) gene fragment^26^ followed by sequencing to determine the species. Only 10 out of 16 samples from infected dogs were successfully sequenced. The six samples left were positive to *Leishmania* sp but not sequenced. The primer sequences are described in Table 1. PCR was performed following previously described cycling conditions: 94°C for 5 minutes followed by 32 cycles at 94°C for 30 seconds, 52°C for 1 minute and 72°C for 90 seconds and a final extension at 72°C for 10 minutes.

**Table 1:** Primers ITS‐1 sequences^27^

Sense	Sequence (5′‐3′)	Size, bp
Forward (LITSR)	CTG GAT CAT TTT CCG ATG	⋯
Reverse (L5.8S)	TGA TAC CAC TTA TCG CAC TT	320

Abbreviations: ITS‐1, internal transcribed spacer‐1; bp, base pairs.

After amplification, the products were purified with MinElute 96 UF PCR purification kit, Qiagen. The purified products were sequenced using the same primers with BigDye Terminator v3.1 Cycle Sequencing Kit (Thermo Fisher Scientific, EUA) according to the manufacturer's specifications, in 96‐well plates using a concentration of 3.2 μM of each primer (forward and reverse) and 1.6 μL of the purified PCR product. The reactions were performed by Fiocruz sequencing platform (RPT01A; Seqüenciamento de DNA, RJ). The obtained sequences were analyzed using *Phred/Phrap/Consed* script and aligned with MEGA 7 software (Gordon D, 2003). A search using the BLAST algorithm (http://ncbi.gov) was carried out to confirm the species.

### 2.10 Detection of amastigote forms of *L. infantum* by ISH

ISH was used only in thymus samples in which the *Leishmania* species was not identified by PCR. In these samples, ISH was used to identify amastigote forms of *L. infantum* by detecting a specific nucleic acid sequence of this parasite. For this purpose, an *L. infantum*‐specific oligonucleotide probe labeled with digoxigenin that targets the minicircle kinetoplast DNA gene of the parasite^28^ was used. Five‐micrometer‐thick sections were cut from the paraffin blocks and mounted on silanized slides. These sections were processed as previously described^29^ using the ZytoFastPlus CISH Implementation Kit AP‐NBT/BCIP1 (Zytovision GmbH, Bremerhaven, Germany). The probe was diluted 1:500 in Hybridization Solution H7782 (Sigma‐Aldrich Co).

### 2.11 Statistical analysis

**Figure 1:**
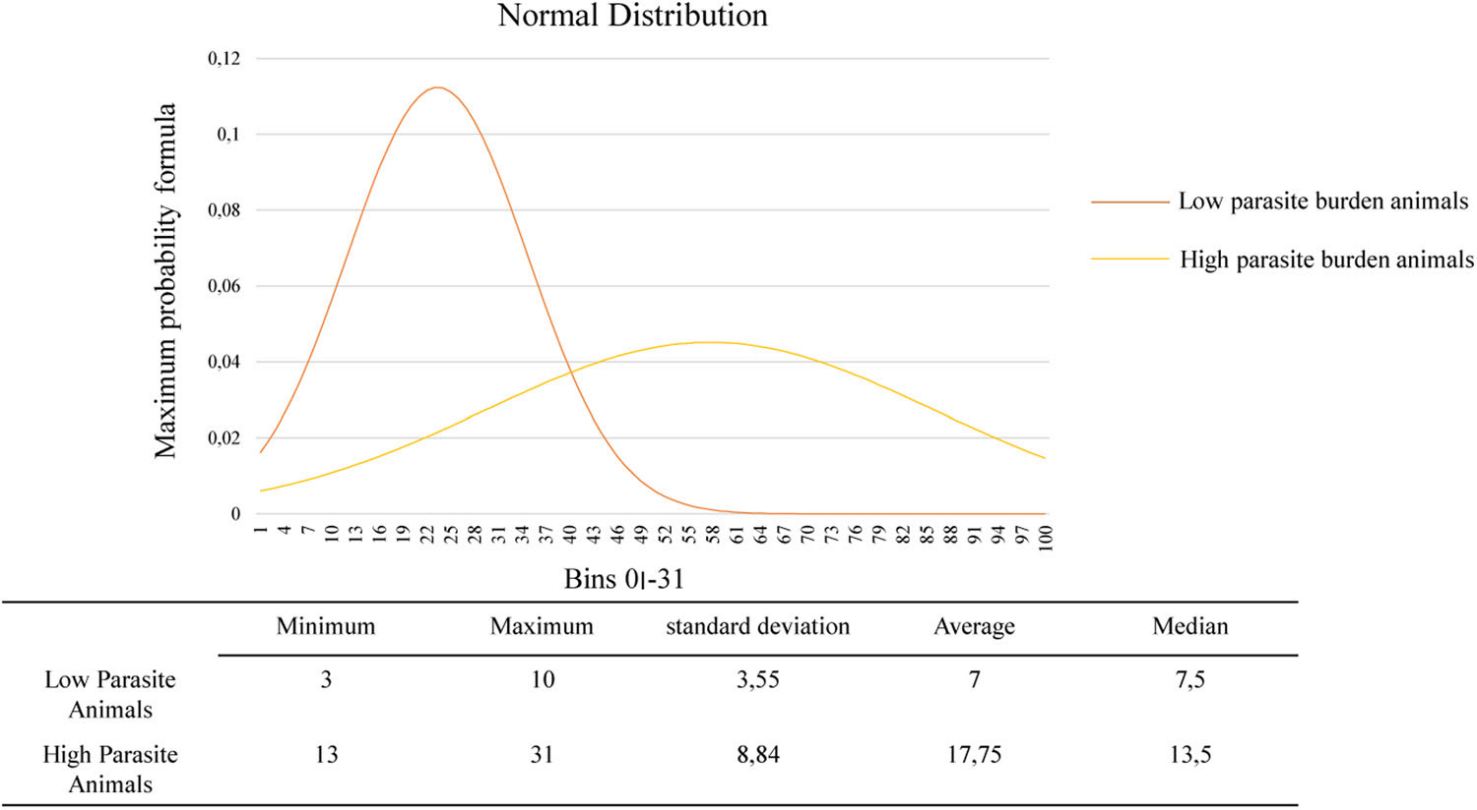
Quantification of parasite load was performed counting the number of amastigotes/mm^2^ of each animal. A Gaussian mixture (Normal distribution) was performed resulting in two groups: low and high parasite load

In the present study, convenience samples were used, and for analysis purposes, the animals were divided into three groups according to clinical signs: without clinical signs, with few clinical signs and with many clinical signs or parasite load: absent, mild to moderate (0.2 to 10 parasitized macrophages), and intense (more than 10 parasitized macrophages). All variables (laminin and fibronectin deposition, cortical and medullary areas, and parasite load‐amastigotes/mm^2^) had normal distribution (*P* > .05) as indicated by Kolmogorov‐Smirnov test in SPSS software. The construction of the Gaussian mixture (Normal distribution) was performed in excel and SPSS 24 software. A table with average, median, standard deviation, and maximum and minimum value of the number of amastigotes/mm^2^ was constructed. The normal distribution and the division into groups according to the intensity of the parasite load (high and low; Figure 1) were performed based on our previous study.^5^ The correlations between the variables (clinical score, cortical/medullary areas, laminin/fibronectin depositions, and parasite load) were performed using the Pearson correlation. The comparisons between groups (high/low parasite load and clinical signs) were then analyzed using a *t*‐independent test.

## 3 RESULTS

### 3.1 Clinical data

Ten of 16 dogs analyzed were males and six were female. The median age was 2.5 (range, 1‐12 years) (Table 2). In the clinical examination, seven dogs had no clinical signs, six had two or more clinical signs and three had one clinical sign (Table 2).

**Table 2:** Description of clinical data and detection of amastigotes in the thymus from *Leishmania infantum*‐naturally infected dogs

Dog	Breed	Age, y	Sex	Clinical signs	Amastigotes in thymus (IHC)	Parasite load/mm^2^	*L. infantum* in SK (LN and SP) and PC
1	Mixed‐breed	3	M	Alopecia	Positive	13	SK(+), LN(+), and SP(+)
2	Mixed‐breed	1	M	Furfuraceous desquamation of skin, thinning, onychogryphosis, and keratoconjunctivitis	Positive	5	SK(+), LN(+), and SP(+)
3	Rottweiler	6	F	Absent	Negative	0	SK(−), LN(−), and SP(−)
4	Mixed‐breed	4	F	Alopecia, lymphadenomegaly Onychogryphosis	Negative	0	SK(+), LN(+), and SP(+)
5	Mixed‐breed	2	F	Absent	Negative	0	SK(−), LN(−), and SP(+)
6	Cane corso	4	F	Thinning	Positive	14	SK(+), LN(+), and SP(+)
7	Cane corso	2	M	Furfuraceous desquamation of skin, alopecia, cachexia, onychogryphosis, keratoconjunctivitis, lymphadenomegaly, and splenomegaly	Negative	0	SK(+), LN(+), and SP(−)
8	Pinscher	4	M	Absent	Negative	0	SK(+), LN(+), and SP(+)
9	Mixed‐breed	3	M	Splenomegaly, lymphadenomegaly	Negative	0	SK(+), LN(−), and SP(−)
10	Mixed‐breed	12	F	Absent	Negative	0	SK(−), LN(−), and SP(+)
11	Mixed‐breed	2	M	Absent	Positive	13	SK(+), LN(+), and SP(+)
12	Mixed‐breed	2	M	Splenomegaly	Positive	03	SK(+), LN(+), and SP(+)
13	Mixed‐breed	5	M	Absent	Negative	0	SK(+), LN(+), and SP(+)
14	Mixed‐breed	1	M	Furfuraceous desquamation of skin, keratoconjunctivitis, lymphadenomegaly, and hepatomegaly	Positive	10	SK(+), LN(+), and SP(+)
15	Mixed‐breed	2	F	Hepatomegaly, skin ulcer, lymphadenomegaly, alopecia, and onychogryphosis	Positive	31	SK(+), LN(+), and SP(+)
16	Mixed‐breed	1	M	Absent	Positive	10	SK(−), LN(+), and SP(+)

Abbreviations: IHC, immunohistochemistry; LN, lymph node; PC, parasitological culture; SK, skin; SP, spleen.

### 3.2 *L. infantum* infecting thymus

**Figure 2:**
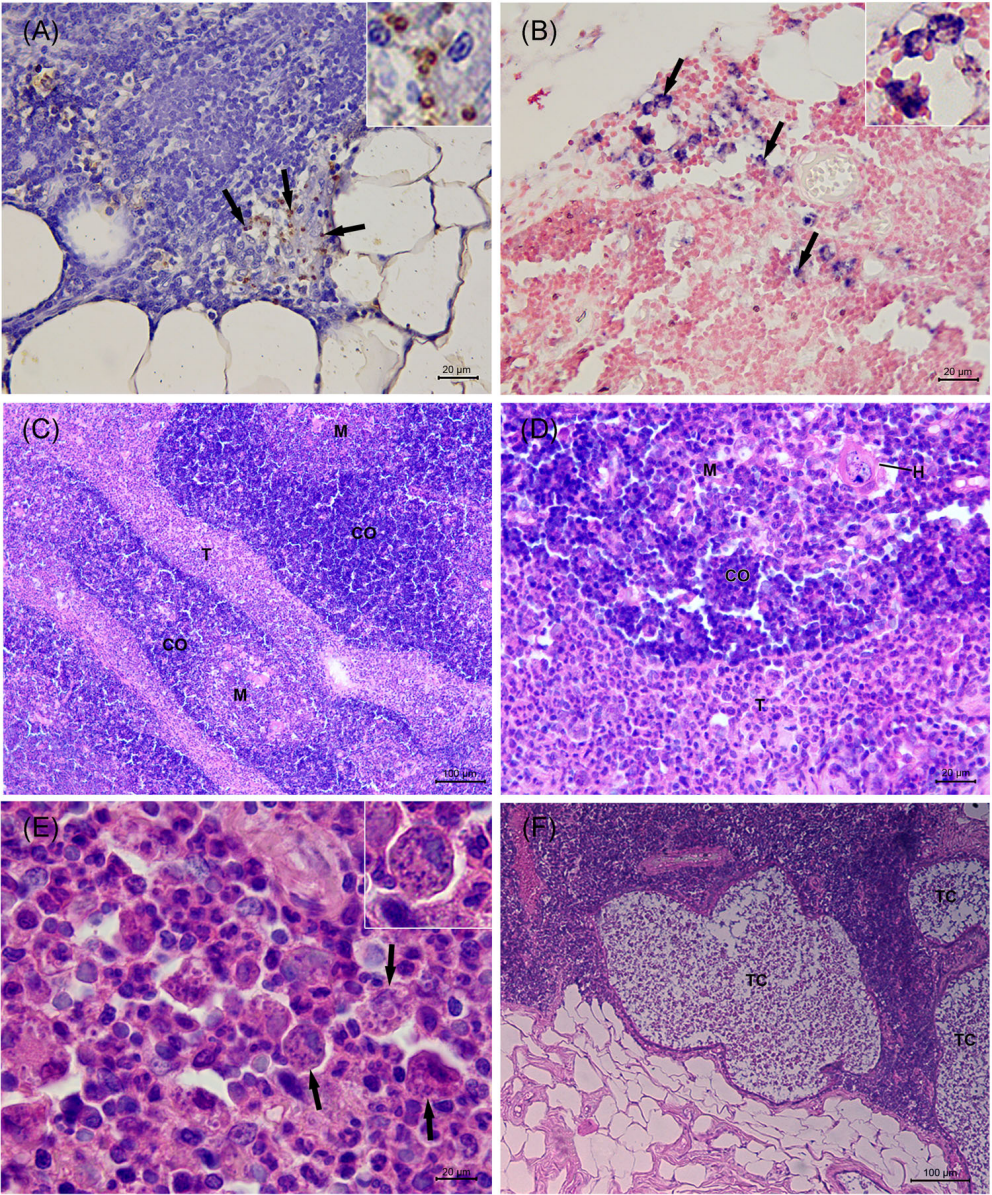
Histopathological evaluation of the thymus from *Leishmania infantum*‐naturally infected dogs. A, Brown‐stained amastigote forms of *Leishmania* spp. (arrow and inset) in the cytoplasm of macrophages (immunohistochemistry). B, Blue‐stained amastigote forms of Leishmania spp. (arrow and inset) in the cytoplasm of macrophages (in situ hybridization). C, Thymitis showing thickening of the interlobular trabeculae due to fibrosis and to an intense pyogranulomatous inflammatory infiltrate (HE). D, Detail of (C) showing an intense pyogranulomatous inflammatory infiltrate in the interlobular trabeculae. E, Detail of (D) showing an intense and poorly formed pyogranulomatous inflammatory infiltrate in the interlobular trabeculae composed mainly by macrophages and neutrophils with few lymphocytes and plasma cells. Several amastigote forms of *Leishmania* spp. (arrow and inset) are observed within the cytoplasm of macrophages (HE). F, Thymic cysts lined by ciliated epithelium in the parenchyma (HE). C, cortex; H , Hassall's corpuscle; HE, hematoxylin and eosin; M, medulla, T, interlobular trabecula; TC, thymic cyst

All 16 dogs tested positive for *Leishmania* sp. DNA by PCR in thymus. After ITS‐1 target amplification, the amplified products from the samples of 10 dogs were identified by sequencing and sequence blast revealed that all DNA samples corresponded to *L. infantum* (Table 3). Amastigote forms within macrophages were detected in the thymic cortex and medulla from eight (50%) dogs by immunohistochemistry (Figure 2A). In the thymus of two dogs that tested positive by PCR, but in which the parasite was not identified at the species level by this technique, ISH was positive for amastigote forms of *L. infantum* (Figure 2B). The thymus parasite load was assessed by immunohistochemistry, and of 16 dogs evaluated, eight showed no label (50%), four dogs (25%) showed mild to moderate parasitism, and four dogs (25%) intense parasitism (Table 2).

**Table 3:** Data description of the sequencing of *Leishmania* internal transcribed spacer‐1 (ITS‐1) gene fragment

Identity animal	Max score	Total score	Query cover, %	*E* value	Degree of similarity sequence, %	Accession	Sequence similar
1	361	361	83	6.00E−96	98	KY658231.1	*L. infantum*
2	424	424	99	7.00E−115	98	KY658235.1	*L. infantum*
3	315	315	74	5.00E−82	96	KY658235.1	*L. infantum*
4	438	438	99	3.00E−119	99	KY658231.1	*L. infantum*
6	442	442	91	2.00E−120	99	KY658231.1	*L. infantum*
7	438	438	82	3.00E−119	99	KY658231.1	*L. infantum*
9	235	235	54	3.00E−58	98	KY658231.1	*L. infantum*
10	353	353	83	9.00E−94	99	FJ555210.1	*L. infantum*
12	207	207	40	1.00E−48	95	FJ497004.1	*L. infantum*
16	385	385	65	4.00E−103	100	KY658235.1	*L. infantum*
IOCL579 (Reference)	479	479	37	5.00E−131	100	KY658231.1	*L. infantum*
IOCL566 (Reference)	468	468	57	6.00E−128	100	KP274863.1	*L. braziliensis*

### 3.3 *L. infantum* infecting other organs

Fifteen (94%) of the 16 evaluated dogs were positive for *Leishmania spp*. in the skin, spleen, or lymph node by parasitological culture (Table 2). The parasite was isolated by culture from spleen, skin and lymph node in 13 (81%), 12 (75%), and 12 (75%) animals, respectively. All these isolates were identified as *L. infantum* by *MLEE*.

### 3.4 Histopathological evaluation

In the thymus of eight dogs positive for *Leishmania* amastigote forms, a granulomatous or pyogranulomatous thymitis was observed: in six dogs (#1, 2, 11, 12, 14, and 16) and two dogs (#6 and 15), respectively. The granulomatous inflammatory infiltrate was poorly delimited and composed mainly by macrophages with few lymphocytes and plasma cells. The pyogranulomatous inflammatory infiltrate was poorly delimited and composed mainly by macrophages and neutrophils with few lymphocytes and plasma cells. The distribution of the inflammatory infiltrate was multifocal or diffuse and it affected cortical and medullar regions. In one dog (#6), the interlobular trabeculae were thickened due to fibrosis and intense pyogranulomatous inflammatory infiltrate associated with many amastigote forms of *Leishmania* within macrophages (Figure 2C‐E). In addition, involution with replacement by connective and adipose tissues was observed in all dogs. Thymic cysts, lined by ciliated epithelium, were found in four dogs (#2, 6, 12, 14) (Figure 2F). In the thymus of the eight dogs in which *Leishmania* amastigote forms were not detected, thymitis was not observed. All of these eight dogs had involution of thymus and thymic cystis was observed in three dogs (#3, 4, and 13).

### 3.5 Analysis of cortex:medulla ratio

**Figure 3:**
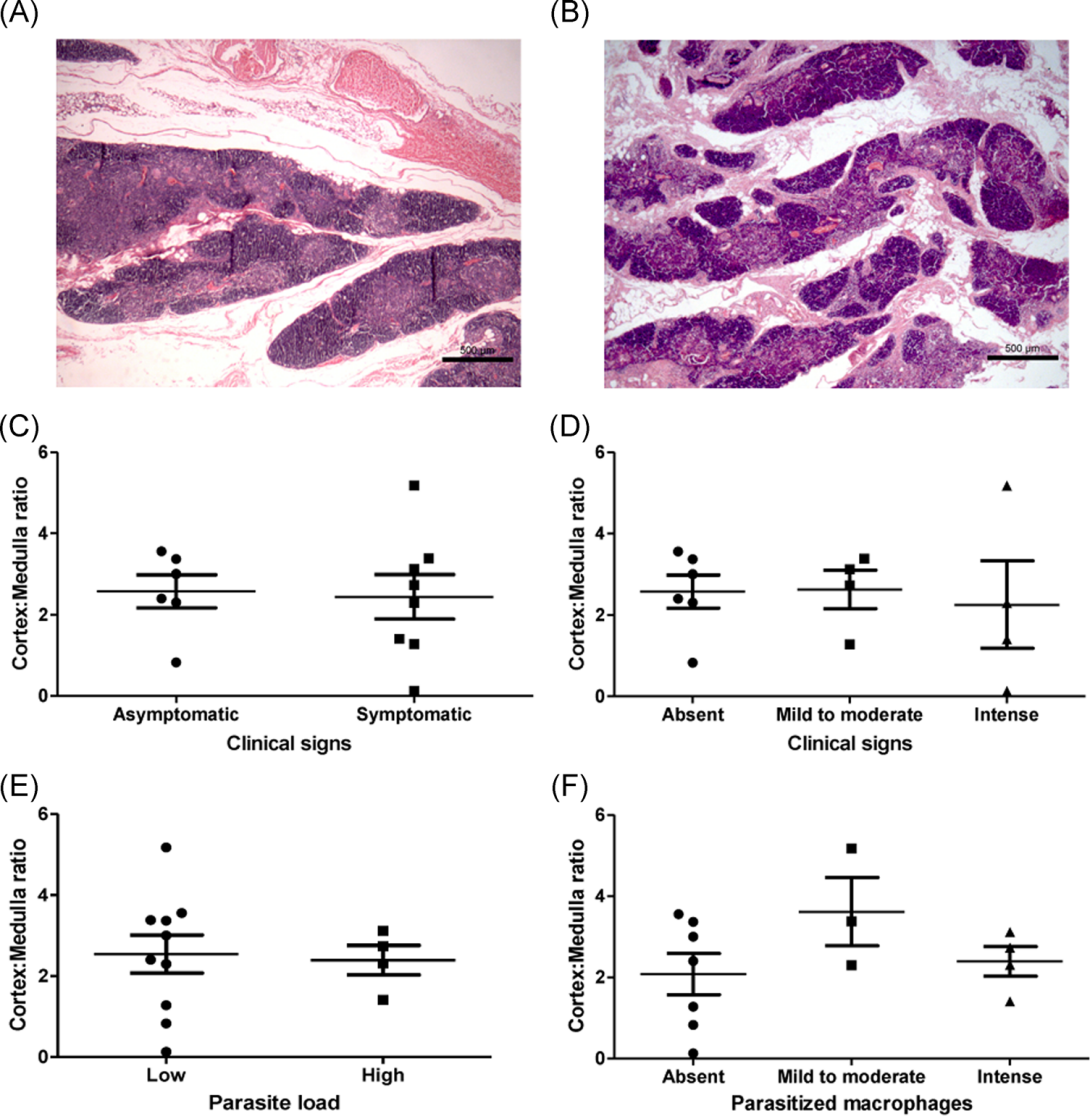
Analysis of the cortex:medulla ratio of the thymus from *L. infantum*‐naturally infected dogs. A,B, Representative images of the cortex:medulla ratio observed in two animals. C,D, Comparison of cortex:medulla ratio between groups according to clinical signs. E,F, Comparison of cortex:medulla ratio between groups according to parasite load

The cortex:medulla ratio was calculated for each dog and varied from 0.13 to 5.18 mm^2^ (mean ± SEM: 2.5 ± 0.35). We did not observe differences in cortex:medulla ratio when groups based on age, clinical signs or parasite load were compared (*P* > .05) (Figure 3).

### 3.6 Analysis of thymic extracellular matrix compounds (laminin and fibronectin depositions)

**Figure 4:**
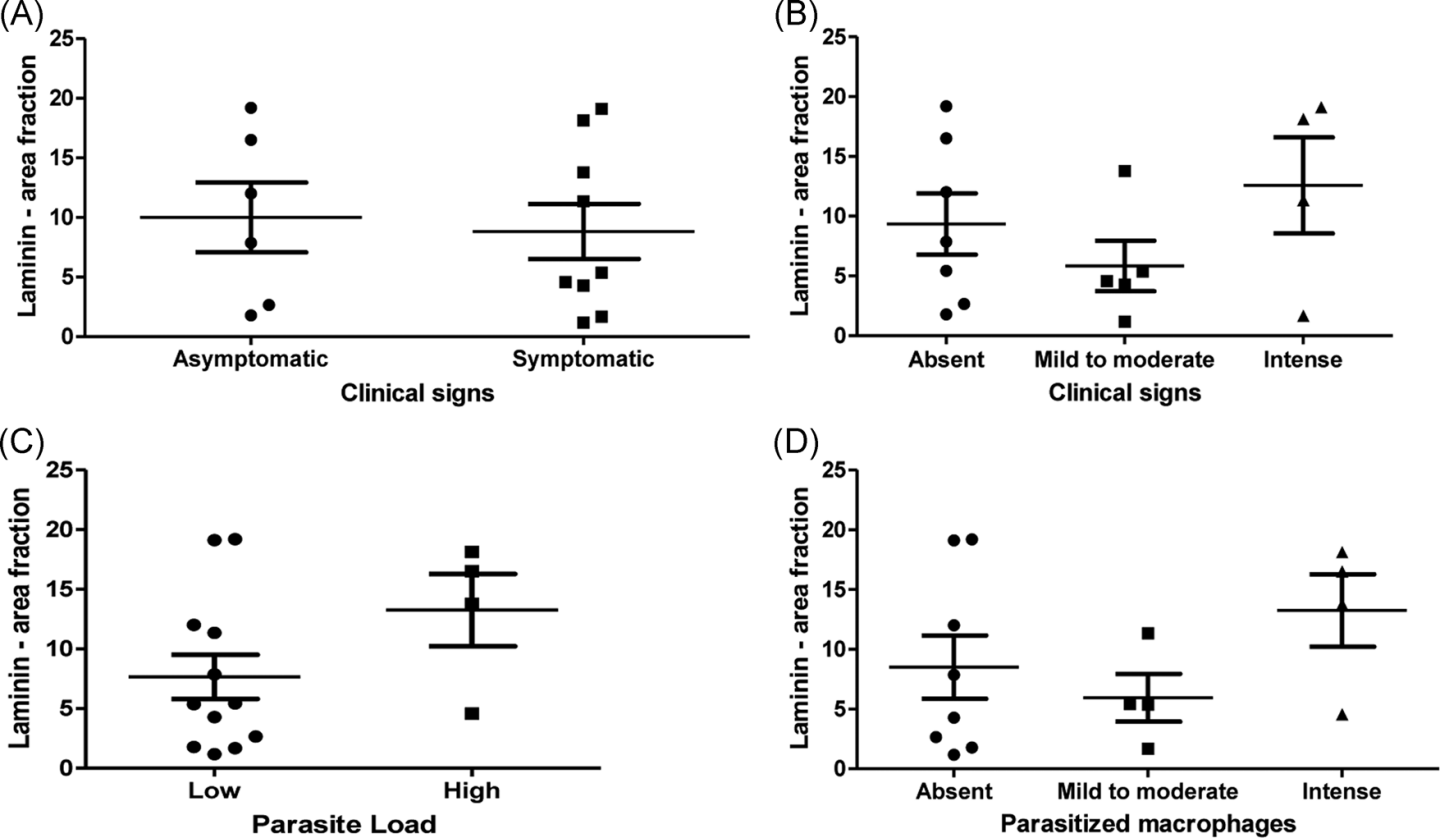
Analysis of laminin deposition in the thymus from *L. infantum*‐naturally infected dogs. A,B, Comparison of laminin deposition between groups according to clinical signs. C,D, Comparison of laminin deposition between groups according to parasite load

**Figure 5:**
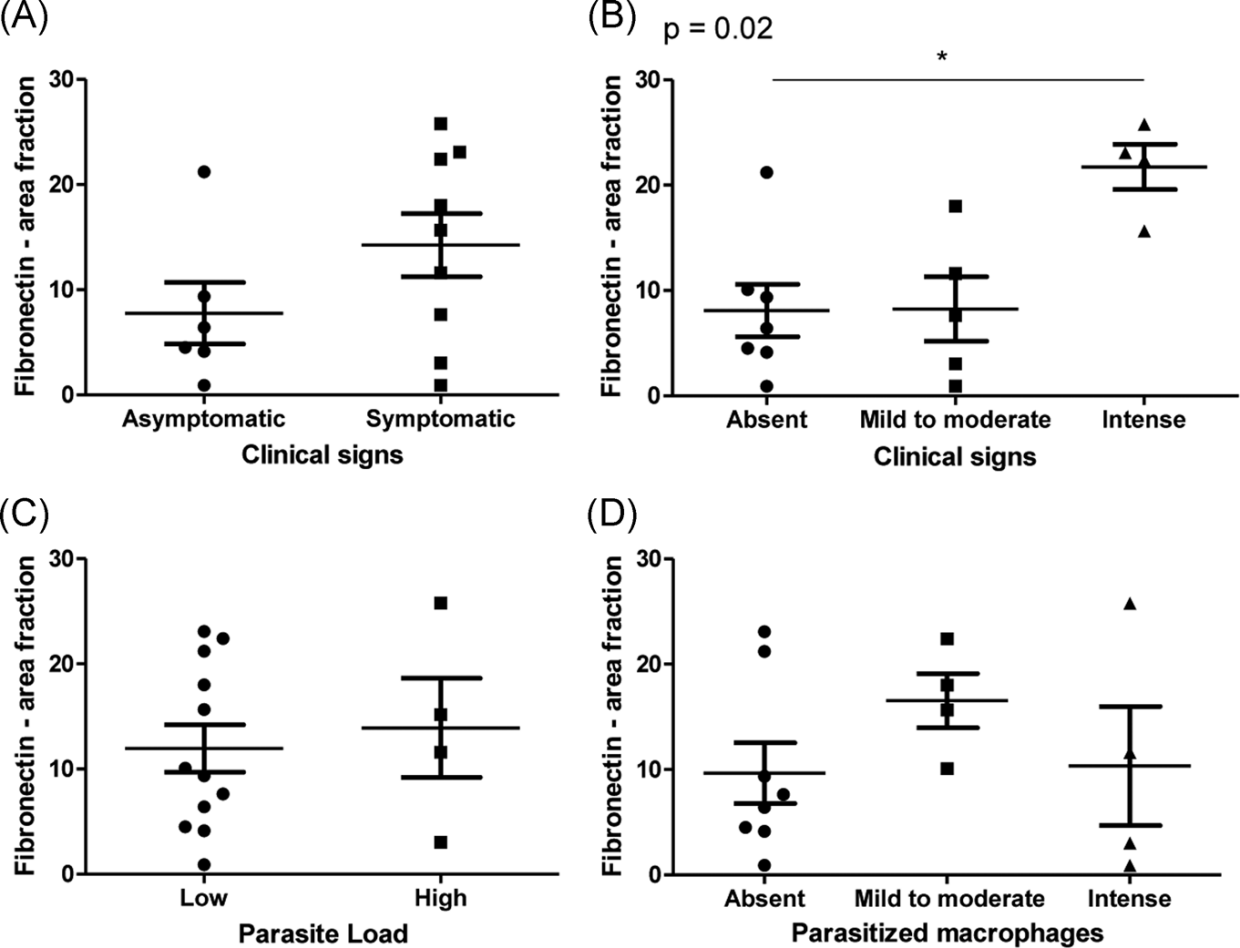
Analysis of fibronectin deposition in the thymus from *L. infantum*‐naturally infected dogs. A,B, Comparison of fibronectin deposition between groups according to clinical signs. C,D, Comparison of fibronectin deposition between groups according to parasite load

**Figure 6:**
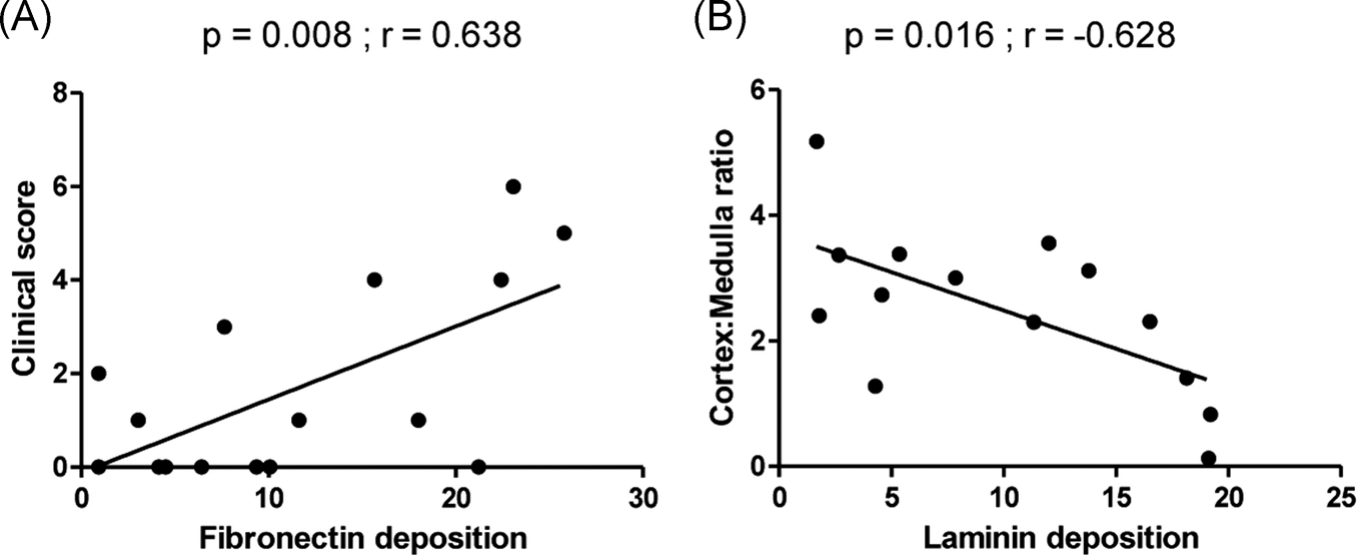
Correlations between (A) fibronectin deposition and clinical score and (B) laminin deposition and cortex:medulla ratio (Pearson correlation)

As we had observed amastigotes in the thymus associated with in situ inflammatory reaction, we decided to quantify some components of thymic extracellular matrix, such as laminin and fibronectin. These molecules act as a scaffold to thymus and play a role in the events of T‐lymphocyte maturation. Fibronectin and laminin depositions were detected in the thymus from all evaluated dogs and varied from 0.92 to 25.80 area fraction (mean ± SEM: 12.44 ± 1.98) and from 1.18 to 19.20 area fraction (mean ± SEM: 9.06 ± 1.66), respectively (Figures 4 and 5). Although we did not observe differences when comparing the area fraction of fibronectin and laminin deposition with the parasite load, we observed that fibronectin deposition was greater in animals presenting more clinical signs (*P* = .028) (Figure 5B). There was a positive correlation between fibronectin deposition and clinical signs (Pearson correlation, *P* = .008; *r* = .638) (Figure 6A). We also observed a negative correlation between laminin deposition and cortex:medulla ratio (Pearson correlation, *P* = .016; *r* = −.628) (Figure 6B).

## 4 DISCUSSION

In the present study, the thymuses of 16 dogs were evaluated for the presence of the parasite *L. infantum*. Herein, we detected Leishmania DNA by PCR ITS‐1 in the thymus from all dogs included in the study and intact amastigotes by immunohistochemistry in 50% of them. The animals showing thymus infection also had parasites in other lymphoid organs such as spleen and lymph nodes. Furthermore, skin infection by *L. infantum* was frequently observed, which suggests that these dogs were potential source of infection for sandy flies.

Histopathological alterations such as granulomatous or pyogranulomatous infiltration were evidenced in the thymuses from eight dogs in which intact amastigotes were visualized in situ. Moreover, dogs presenting more clinical signs showed higher fibronectin depositions in thymus. Altogether, the data suggest the thymus as a site for parasite colonization. Moreover, the alterations in thymic microarchitecture could be associated with inflammatory reactions induced by the parasite in the organ and may influence the antiparasite immune response and disease progression.

The thymic microenvironment is formed mainly by fibronectin and laminin.^30^ The bone marrow‐derived T‐cell precursors enter in the thymus by the cortical zone and travel through the thymic microenvironment to finish their maturation.^30^, ^31^, ^32^ Cell migration is crucial for intrathymic T‐cell differentiation induced by chemokines and extracellular matrix components such as fibronectin.^30^ Parasitic infections lead to thymic changes that may affect the immune response of animals.^33^, ^34^, ^35^ In a previous study, amastigotes were evidenced in mice experimentally infected with *L. infantum*, and the thymuses of those animals exhibited increased weight and significant alterations in chemokine levels.^35^ Morrot et al^33^ studying an experimental acute infection by *Trypanosoma cruzi*, observed massive thymocyte depletion and abnormal release of immature CD4(+)CD8(+) cells to peripheral lymphoid organs, where they acquired an activated phenotype similar to activated effector or memory T cells. They suggested that, in this model, these cells escaped from the negative selection process, and some of them became potentially autoimmune.^33^ They also observed atrophy of mesenteric lymph nodes, in contrast with lymphocyte expansion in spleen and subcutaneous lymph nodes, illustrating a complex and organ‐specific dynamics of lymphocyte subpopulations.^33^ Herein, we observed inflammatory reaction and alterations in fibronectin depositions in infected dogs. Since the cortical region is the area where immature lymphocytes migrate while they receive the stimuli for their maturation, the data suggest that *Leishmania* infection could modify the regular functionality of thymus. In fact, lymphopenia, splenomegaly, and lymphadenopathy have been described in canine visceral leishmaniasis (CVL), as well as microarchitecture disruption of both spleen and lymph nodes.^8^, ^36^, ^37^ Although the presence of amastigotes in spleen and lymph nodes can produce local inflammatory alterations, based on our data, we cannot discard the possible contribution of immature lymphocytes released from the thymus to the described tissue alterations. Therefore, other analyses should be done to verify this hypothesis.

In our study, the animals were in the adult phase and we observed involution of thymus in all of them. In dogs, the complete development of thymus occurs at 2 months of age, entering into a process of involution after 6 months of age, but maintaining its activity during the animal's life and acting as a modulator of other lymphoid organs.^18^ From 1‐year‐old, the adipose tissue becomes more evident with small islands of remaining thymic tissue,^18^ which may persist until the old age. In fact, we observed the islands of remaining thymic tissue in one 12‐year‐old dog. These islands had all cell populations as observed in the thymus from young animals but in smaller numbers.^18^ So, it is reasonable to think that due to infection by *Leishmania* and consequent tissue damage observed in the thymus, the maturation of lymphocytes could be altered in these thymic islands and might contribute to the immunopathology of secondary lymphoid organs in CVL. Although the reminiscent thymus maintains thymocyte maturation over the whole life of the animal, the effect of thymic infection in the course of leishmaniasis would be more severe in pups, since there is intense thymus activity and immunological maturation in young animals. Thus, studies focused on the thymus of young dogs would better‐clarify this hypothesis.

Regarding the alterations observed in thymic extracellular matrix compounds, fibronectin interconnecting segment domain can be recognized by receptors expressed in macrophages favoring the invasion of parasites and reducing the activation of macrophages.^38^, ^39^, ^40^, ^41^ In fact, the thymus is an organ that harbors a significant number of TGF‐β (transforming growth factor‐β)‐producing macrophages that act as professional scavengers of apoptotic cells resulting from the regular process of negative selection.^42^, ^43^ Due to selection processes, 95% of all the thymocytes formed die and are submitted to clearance by macrophages.^44^ Although classically activated macrophages (M1‐NOS2 and TNF‐α [tumor necrosis factor‐α] producer cells) play a role in parasite killing,^45^ the professional scavenger macrophages observed in thymus could act as a site for *Leishmania* persistence. In fact, we previously observed F4/80+ cells colocalizing with parasites in the thymus of *L. infantum*‐infected mice.^35^ In the infected dogs, the amastigotes were observed inside macrophages located at the cortical and medullary regions.

The higher expression of extracellular matrix molecules such as laminin, collagen, and fibronectin in animals with more clinical signs has been demonstrated in lymphoid organs such as spleen and lymph node, and also in the liver.^8^, ^9^, ^14^, ^37^, ^40^ In our study, we observed a positive correlation between clinical score and fibronectin deposition. This data suggests a remodeling process of the thymic extracellular matrix probably due to the inflammatory process induced by the presence of the parasite in this organ. The parasite disseminates through various organs, causing inflammatory process, extracellular matrix changes and tissue damage leading to organic dysfunctions, and consequently, the appearance of clinical signs. However, further studies should be performed to elucidate this hypothesis.

The lack of correlation between fibronectin expression and cortex/medulla ratio can be explained by the large variability in measurements of these areas due to the age differences of the evaluated dogs. There are several factors that may influence the thymus histological patterns: diabetes,^46^ infections,^34^ malnutrition,^35^ and age.^16^, ^18^ It is well‐described that the thymus suffers involution throughout the life of the animal accompanied by the reduction of the cortex.^16^, ^18^ Laminin, in turn, correlated inversely with the cortex/medulla ratio. Laminin is more concentrated in the medullary region, and therefore, the larger the cortical area, the lower the laminin expression. This correlation probably occurred due to age‐related differences but not to the infectious process itself.

Taken together, these results demonstrate that the thymus of dogs can be parasitized by *L. infantum*, which may generate inflammatory reactions leading to alterations in thymic microarchitecture. These alterations probably compromise the maturation of T lymphocytes in the thymus favoring the failure of the immune response in infected dogs.

## CONFLICT OF INTERESTS

The authors declare that there are no conflict of interests.

## AUTHOR CONTRIBUTIONS

RCM, FNM, and RP conceived and designed the research; AVAS, TLS, FBF, AAVMJ, LCF, CPBF, and PC performed the experiments; AVAS, TLS, RP, RCM, and FNM prepared the figures and wrote the manuscript; AVAS, TLS, RCM, and FNM analyzed the data; AVAS, T.L.S, RP, RCM, and FNM edited the manuscript; and AVAS, TLS, FBF, AAVMJ, LCF, CPBF, PC, RP, RCM, and FNM revised and approved the final manuscript.

## Data Availability

The data that support the findings of this study are available from the corresponding author upon reasonable request.
